# Supplementary material for: Exploring the sustainability of virtual care interventions: A scoping review
Source: PLOS Digit Health. 2025 Jun 11;4(6):e0000893. doi: 10.1371/journal.pdig.0000893 (PMC12157087; doi:10.1371/journal.pdig.0000893)
Supplement: Attachment — Submitted filename: Response to Reviewers.docx [file pdig.0000893.s002.docx]

Dear Editors and reviewers,

We would like to thank you and the reviewers again for the thoughtful feedback, which has enabled us to improve our manuscript. We have addressed each comment from the reviewers below in a table format. The requested changes are indicated in track changes within the manuscript document. Please let us know if you have any questions.

| **Reviewer comment** | **Authors’ response** | **Manuscript page and line number** |
| --- | --- | --- |
| Many words are often used to describe different or same things related to virtual care. Regarding the additional provided document “Search Strings”, I fear that the authors have lost many valuable papers that may use for example “video consultation/consulting” or “video appointments” instead of “virtual care”. Adding “remote” or “video” might provide more papers to use. In the same vein, as the paper mentioned as well, the concept of sustainability may be part of a paper without using the search terms that appear in the search term document. Generally, 21 papers seems to be too few papers in the given context, hence my ideas to improve the search strategy.  I am also confused by the stated research question on page 6. To find “what is known” about sustainability in virtual care does only very broadly match the mentioned goals of a) providing a conceptual framework and b) finding factors that influence sustainability. In the same vein, I became more confused when reading the paper on whether the authors want to keep a narrow or wider view on sustainability. Their exclusion criteria regarding sustainability may limit potential candidates too much. As a reader “do not conceptualize/describe/define sustainability” is not entirely clear to me how much gets lost. | Thank you for this feedback. While a librarian was consulted in the development of the search strategy, and the rationale for not including the terms “video” and “remote” was that the term telehealth has been used in the literature to encompass phone, video, remote monitoring, video appointments, and video consultations, we welcome the feedback to enhance the robustness of the search. The intention of this research was to understand sustainability as it specifically relates to virtual care interventions. Therefore to be included, papers had to explicitly define, describe, or conceptualize the term sustainability. Many papers were excluded as they only mentioned sustainability in the context of "program x was sustained for x time period" but did not describe what was considered as sustained (i.e., the virtual care intervention itself? The benefits of the intervention? Was the intervention changed or modified during the sustained time period? Did changes constitute a modified version of the intervention or a new intervention?). We have re-run the search and included the terms “video” and “remote”. The re-run search yielded a total of 1413 new papers. 687 of these were duplicates of previously screened studies; 533 were excluded at title/abstract screening, 24 at full- text screening. Of the 169 remaining papers, 127 a did not define sustainability, and 38 focused on implementation. The 4 newly included studies did not alter the findings of this work but have been incorporated into the data tables and analysis. | Page 10, line 200  Pages 19 and 20 |
| At the end of the paper, page 30 line 379-380, the authors mention the study’s addition with respect to covid in the setting. I fear however that the paper is not clear enough on Covid in general. The conclusion of covid being the driver for innovation and creativity does not match the overall paper’s mentioning of covid and I was surprised to read that sentence in the conclusion. The paper may benefit from more clear explanations regarding this inside the main text. For now, I am confused on how the paper tries to argue with regards to covid. | Thanks, we have added details for clarity. | Page 4, lines 97-102 |
| To my understanding Fidelity and adaptability open the paper section on how factors influence sustainability of virtual care. To the reader this section could benefit from more structure and introduction. After the conceptual framework, a section of influencing factors marks the second big section in this paper according to the abstract. More introduction and general larger frame for this chapter may help convey the findings from the scoping review. | Thanks, we have revised the introduction to the results section for clarity. | Page 22, Lines 220-222 |
| Minor comments  a) Typo in flow chart “conceputalize”  b) Page 3 line 71 to 74 sentence structure confusing  c) Page 3 line 75 hospitals s missing  d) Page 4 line 94 how can non-essential surgeries backlog be solved by virtual care needs more explanation  e) Page 8 line 170 “kappa score” not clear to me  f) Page 23 line 239 “paired” not clear  g) Page 23 line 253 and 254 confusing sentence  h) Page 25 line 290-292 confusing sentences  i) Page 29 line 350 does not match previous sentence part  j) Page 31 line 405 and 406 two times “question” | Thank you. These corrections and clarifications have been made. | Page 3, lines 72,75  Page 4, lines 94, 95  Page 8, line 178  Page 10, line 207  Page 25, line 251, 265  Page 27, line 300  Page 31, line 362  Page 33, lines 417, 418 |
| L34-54: the conceptual framework could be more clearly outlined in the results section of the abstract. | Thanks, we have added details for clarity. | Page 2, lines 43-45 |
| L56-68: consider briefly defining what a conceptual framework is and why it is used in the author summary for the benefit of non-experts. | Thanks, we have added a brief description of a conceptual framework. | Page 3, lines 60 |
| L124-129: build a stronger argument for the factors considered in the conceptual framework. Why are fidelity and adaptability considered to be important? Why does it make sense to develop a conceptual framework focus only on these two factors? | Thanks, we have added details for clarity. | Page 6, lines 137-140 |
| L139-141: consider linking the research question to the review objectives (e.g., in asking this question, a conceptual framework will be developed, etc.) | Thanks, we have added details for clarity. | Page 6, lines 154-156 |
| L154-156: these criteria could be made clearly – particularly this part: “…or reference to the study authors’ provided 156 definition or conceptualization (including contributing factors to) of the term sustainability”. | Thanks, we have revised this sentence for clarity. | Page 7, line 170 |
| L168-172: state how many reviewers extracted data. | Thanks, we have included this information. | Page 8, line 189 |
| L181-190: how was the data summarised to get a descriptive understanding and develop patterns? What kind of software/method was used to organise data for analysis? If an established method was used, state this. How many team members were involved in this process? Etc. | Thanks, we have added these details. | Page 9, lines 194-196 |
| L390-392: The term “disruptive innovation” is used here which represents the general focus of this review which is on the novel application of technology to virtual care. To counterbalance this theme, I suggest including a paragraph that acknowledges structural factors at play that force the use of such technologies (e.g., understaffed facilities, poor transport to care facilities, etc.). It could link in with the point made in the introduction regarding when it is and is not appropriate to use such technologies. I see that the feasibility of these technologies is raised as a question for future researchers to answer to this point could also link in with that | Thanks, we have added details for clarification. | Pages 32 and 33, lines 413-423 |
